# Mitosis-specific phosphorylation of amyloid precursor protein at Threonine 668 leads to its altered processing and association with centrosomes

**DOI:** 10.1186/1750-1326-6-80

**Published:** 2011-11-23

**Authors:** Monique Judge, Lisa Hornbeck, Huntington Potter, Jaya Padmanabhan

**Affiliations:** 1 Department of Molecular Medicine, University of South Florida, 12901 Bruce B. Downs Blvd., Tampa, FL-33612, USA; 2 USF Health Byrd Alzheimer's Institute, 4001 E. Fletcher Ave., Tampa, FL-33613, USA; 3 Suncoast Gerontology Center, USF Health Byrd Alzheimer's Institute, 4001 E. Fletcher Ave., Tampa, FL-33613, USA; 4 Florida Alzheimer's Disease Research Center, USF Health Byrd Alzheimer's Institute, 4001 E. Fletcher Ave., Tampa, FL-33613, USA

**Keywords:** Amyloid precursor protein, cell cycle, mitosis, kinases, APP phosphorylation, amyloid processing

## Abstract

**Background:**

Atypical expression of cell cycle regulatory proteins has been implicated in Alzheimer's disease (AD), but the molecular mechanisms by which they induce neurodegeneration are not well understood. We examined transgenic mice expressing human amyloid precursor protein (APP) and presenilin 1 (PS1) for changes in cell cycle regulatory proteins to determine whether there is a correlation between cell cycle activation and pathology development in AD.

**Results:**

Our studies in the AD transgenic mice show significantly higher levels of cyclin E, cyclin D1, E2F1, and P-cdc2 in the cells in the vicinity of the plaques where maximum levels of Threonine 668 (Thr668)-phosphorylated APP accumulation was observed. This suggests that the cell cycle regulatory proteins might be influencing plaque pathology by affecting APP phosphorylation. Using neuroglioma cells overexpressing APP we demonstrate that phosphorylation of APP at Thr668 is mitosis-specific. Cells undergoing mitosis show altered cellular distribution and localization of P-APP at the centrosomes. Also, Thr668 phosphorylation in mitosis correlates with increased processing of APP to generate Aβ and the C-terminal fragment of APP, which is prevented by pharmacological inhibitors of the G1/S transition.

**Conclusions:**

The data presented here suggests that cell cycle-dependent phosphorylation of APP may affect its normal cellular function. For example, association of P-APP with the centrosome may affect spindle assembly and cell cycle progression, further contributing to the development of pathology in AD. The experiments with G1/S inhibitors suggest that cell cycle inhibition may impede the development of Alzheimer's pathology by suppressing modification of βAPP, and thus may represent a novel approach to AD treatment. Finally, the cell cycle regulated phosphorylation and processing of APP into Aβ and the C-terminal fragment suggest that these proteins may have a normal function during mitosis.

## Background

The major pathological characteristics of Alzheimer's disease are the presence of neuritic plaques and neurofibrillary tangles (NFT) in the affected areas of the brain [1-3]. In addition, AD brains show neuroinflammation and neuronal loss, which is associated with aberrant expression of cell cycle regulatory proteins [4-8]. The cause or the function of the increased levels of cell cycle regulatory proteins in post-mitotic neurons is not clearly understood. Studies by different groups suggest that fully differentiated neurons in adult brains emerge from quiescence and attempt to re-enter the cell cycle under pathological conditions [4,8-23]. This apparent upregulation of cell cycle regulatory proteins in neurons, along with the findings that the inhibitors of cell cycle activation protect neurons from undergoing apoptosis, led to the hypothesis that inappropriate attempts by neurons to re-enter the cell cycle may lead to neurodegeneration and apoptosis [6,12,24-32]. In addition to neuronal loss, it is possible that dysregulation of the cell cycle may lead to cell cycle-dependent modifications in the amyloid precursor protein (APP) and tau, the two major proteins associated with AD, favouring plaque and tangle formation and neurodegeneration in the AD brains.

APP is a single transmembrane protein that is sequentially cleaved by β and γ- secretases to generate the Aβ peptide, which gets deposited extracellularly to form plaques and vascular amyloid deposits [33]. Mutations in APP and presenilin 1 (PS1) are associated with increased generation of Aβ and increased pathology development in AD [34]. In addition to the accumulation of Aβ into amyloid, studies in neurons have shown that Aβ peptides can induce cell cycle activation and neuronal apoptosis [35]. Expression of a mutant form of APP or PS1, as well as treatment with Aβ, have been shown to induce chromosome mis-segregation and aneuploidy in cells [36,37], which indicates aberrant cell cycle activation under these conditions. Studies conducted in two different AD mouse models have shown an upregulation of cell cycle regulatory proteins in glial cells [38] and neurons [39]. Thus, cell cycle deregulation may influence both neuronal and glial functions, and a keen analysis of the cell cycle-dependent changes in these cells may reveal the significance of the upregulated expression of cell cycle markers in AD brains. Mice generally do not show much neuronal loss, but it is possible that the upregulation of cell cycle regulatory proteins may mediate synaptic loss and neurodegeneration by inducing modifications in tau and APP. Here we analyzed the specific effects of cell cycle activation on APP modifications.

APP is phosphorylated by multiple kinases, which affects its proteolytic processing, trafficking, and protein-protein interaction [40-48]. We tested the hypothesis that cell cycle activation can affect APP modifications and plaque development, using in vitro cultured cells and transgenic mice. The studies presented here show that transgenic mice expressing mutant APP (APP_V717F_) and PS1 (PS1_M146L_) show an increase in the levels of cell cycle regulatory proteins which is associated with induction of APP phosphorylation at Thr668 and formation of Aβ and phosphorylated C-terminal fragment of APP. Experiments conducted in H4 neuroglioma cells overexpressing APP confirmed that this phosphorylation is mitosis-specific and can be inhibited by G1/S transition inhibitors, which prevent Aβ generation. A role for G1/S specific inhibition was further determined by inhibition of P-APP formation by siRNA to cdk-2. This observation, along with our finding that P-APP co-localizes with MPM2 at centrosomes in mitotic cells suggests that mitotic mechanisms may influence AD pathology by not only affecting APP phosphorylation and Aβ generation, but also by enabling it to have a role in spindle assembly and cell cycle regulation. Thus, APP may act as a cell cycle inducer under mitotic conditions and might play a feed forward role in pathology development in AD.

## Results

### Upregulation of cell cycle regulatory proteins in AD transgenic mice

**Figure 1:**
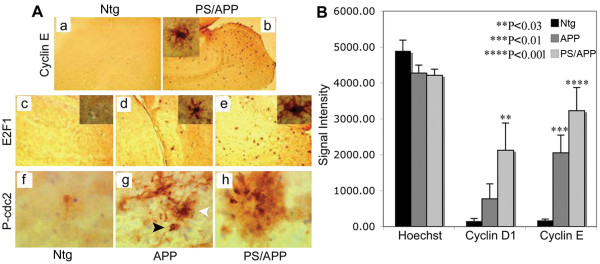
**Increased expression of cell cycle regulatory proteins in AD transgenic mice**: A) Cyclin E, E2F1 and P-cdc2 levels are upregulated in transgenic mice expressing APP and PS/APP: Brain sections from Ntg normal mice (a, c, f) were compared to those from transgenic mice expressing APP (d, g) and PS/APP (b, e, h) using cyclin E (upper panel), E2F1 (middle panel) or P-cdc2 (lower panel) antibodies. Images (a-e, 5× and f-h 20×) were taken using a Nikon E1000 microscope and analyzed using Image-Pro Plus software. The P-cdc2 and images in the inset show magnified images (20×) of the plaques to visualize the cells. We found that the cells surrounding the plaques were positive for cyclin E, E2F1, and P-cdc2, and it appears that both neurons (black arrow head) and glia (white arrow head) were positive for P-cdc2. 'Secondary antibodies only' control did not show any specific staining of the sections (data not shown). B) Quantitative analysis of cyclin D1 and E expression in APP and PS/APP transgenic mice brains: Brain sections from Ntg and mice expressing APP and PS/APP were stained using a monoclonal cyclin D1 or a polyclonal cyclin E antibody and nuclei visualized using Hoechst. The signal intensity was measured using Image J, image processing and analysis program. The signal strength was compared to that with Hoechst nuclear staining from each section to avoid mouse-to-mouse variation. The means of results from six independent mice are shown with standard error bars and P values. While APP mice showed a significantly higher level of only cyclin E compared to cyclin D1, PS/APP mice showed higher levels of both cyclin D1 and E levels compared to Ntg.

**Figure 2:**
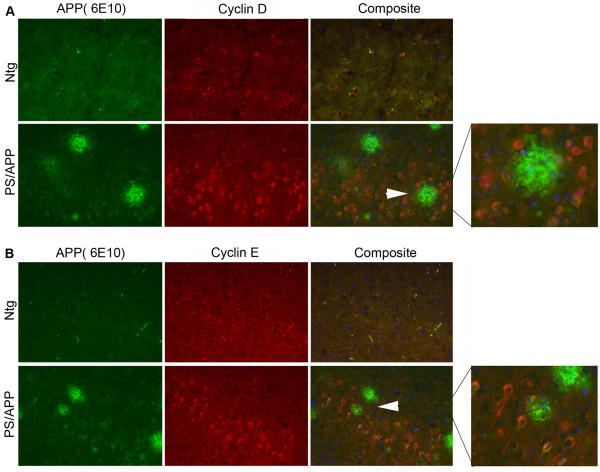
**AD transgenic mice show increased expression of cyclin D and cyclin E in neurons**: Brain sections from 10 month old Ntg and PS/APP mice were co-stained using **A) **monoclonal 6E10 and polyclonal cyclin D1 or **B) **6E10 and polyclonal cyclin E antibodies. Staining was visualized using Alexa fluor 488 (APP and Aβ, green) and Alexa fluor 594 (red) and analyzed under a Zeiss microscope using AxioVision Rel 4.8. The images were taken at 20× magnification. The composite image shows staining with Hoechst, cyclin, and 6E10 antibodies. The area indicated by arrows is enlarged and shown on the right to clearly see the positive staining in neurons.

Atypical expression of cell cycle regulatory proteins has been shown primarily in neurons of AD brains. Studies in different mouse models of AD showed upregulation of cell cycle regulatory proteins with some variation in the observations; while one study showed upregulation of cyclins D1, B and E in astrocytes with cdk4 nuclear translocation [38], another study showed upregulation of PCNA and cyclin A in neurons [39]. Because there is variation in cell cycle protein expression in different AD transgenic mice, we tested the transgenic mice that we used in our studies for changes in the reported cell cycle regulatory proteins. The brains from 12 month old mice expressing mutant human *APP *(V717F), mutant *PS1 *(M136L), a combination of these two transgenes, and age matched non-transgenic (Ntg) controls were analyzed by quantitative immunohistochemistry using specific antibodies to cyclin D1, cyclin E, E2F1, P-cdc2, and cdc2. Significant increases in cyclin D1, cyclin E, P-cdc2, and E2F1 were observed in the PS/APP double transgenic mice (Figure 1A and 1B). Mice expressing APP alone showed a smaller increase in the level of these cell cycle regulatory proteins compared to the double transgenic mice (Figure 1A, d compared to 1A, e), possibly due to the differences in the transgene expression and the fact that PS/APP mice develop pathology at an earlier age compared to APP expressing mice. E2F1 and cyclin E stained cells surrounding the plaques (Figure 1A, inset b, d, and e) appeared to have glial-like morphology, whereas P-cdc2 appeared to stain both neuronal and glial-like cells (Figure 1A, g: black arrow head - neuron & white arrow head - glia). We did not observe any change in the level of staining with a non-phospho-cdc2 antibody. Figure 1B shows the quantitative analysis of cyclin D1 and E levels in APP and PS/APP mice compared to Ntg mice. Co-immunostaining analysis of brains from 10 month old PS/APP mice with cyclin D1 or cyclin E and 6E10 antibodies showed increased levels of these cell cycle regulatory proteins in neurons in the brains (Figure 2A and 2B). Studies from other groups have shown that Aβ can induce neurodegeneration through activation of cell cycle dependent mechanisms [49,50]. Further studies are necessary to determine whether the increased expression of cell cycle regulatory proteins in neurons is brought about by increased levels of Aβ in the AD transgenic brains. It is possible that the cell cycle activation and Aβ generation are regulated in a feed forward manner, with cell cycle activation inducing Aβ production and Aβ in turn inducing cell cycle deregulation.

### Phosphorylation of APP at Thr668 in mice expressing AD transgenes

**Figure 3:**
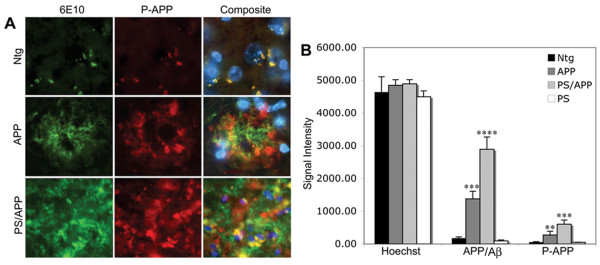
**APP overexpressing mice show increased levels of Thr668 P-APP**: Brain sections from Ntg, APP, and PS/APP mice were immunostained using monoclonal 6E10 antibody and polyclonal Thr668 specific P-APP antibody. **A: **Representative sections from Ntg (top row), APP (middle row), and PS/APP (bottom row) mice stained with a 6E10 antibody (left column), Thr668 P-APP antibody (middle column) and composite image (right column) with Hoechst showing nuclear staining. Magnification: 40×. **B: **Quantification of the intensity of 6E10 and P-APP staining in Ntg, APP, PS/APP and PS mouse brains. Both APP and PS/APP mice showed significantly higher levels of APP and Thr668 P-APP with more intensity in mice expressing both PS and APP transgenes compared to APP alone. The Ntg and PS expressing mice showed low levels of APP and P-APP.

APP is a transmembrane protein and is phosphorylated by several kinases including the cell cycle-dependent kinase cdc2 [45]. Phosphorylation of APP seems to enhance its proteolytic processing and altered localization [42,43]. In order to determine the phosphorylation levels of APP in cells associated with AD pathology, we performed immunohistochemical and western blot analysis of brain samples from mice expressing PS1 or APP alone or together and compared the results to that from age-matched Ntg mice. Co-staining with the Aβ antibody (6E10) and Thr668 specific P-APP antibody (P-Thr668-APP) showed that significantly higher levels of P-APP and Aβ (6E10 positive) associate with the plaques in AD mice (Figure 3). The Aβ staining (6E10) was localized mostly to the plaque cores with some diffuse staining around the plaques whereas P-APP was distributed towards the periphery of the plaques.

**Figure 4:**
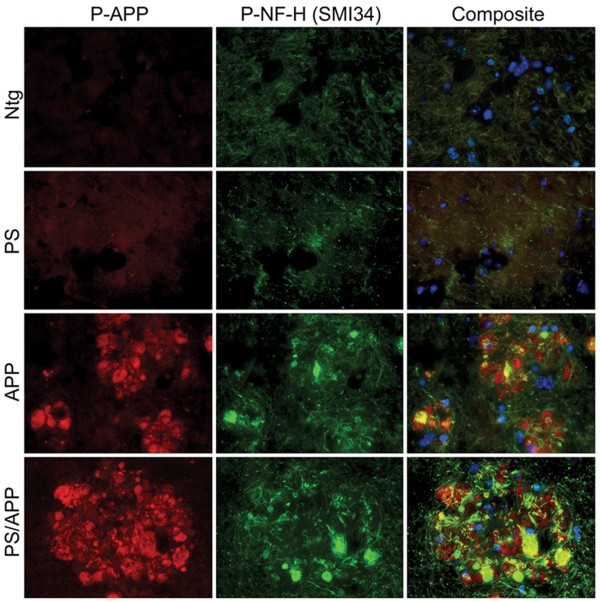
**Thr668 P-APP antibody co-localizes with phospho-neurofilament NFH antibodies in the plaques**: Brain sections from Ntg, PS, APP, and PS/APP mice were analyzed with Thr668 P-APP and monoclonal P-NFH (SMI34) antibodies and visualized using Alexafluor 594 and 488 respectively. Nuclei were visualized using Hoechst stain. Magnification: 40×.

The staining pattern observed with Thr668 P-APP antibody around the plaques suggested that P-APP is accumulating in dystrophic neurites. In order to confirm this, we performed co-staining of the sections with antibodies specific for P-APP and phosphorylated neurofilament H protein (NF-H, SMI34) (Figure 4). SMI34 stains neurofilament proteins when phosphorylated, and it has been shown to stain NFTs and dystrophic neurites [51]. We found co-localization of SMI34 and P-APP in the areas surrounding the plaques in APP and PS/APP mice (Figure 4, rows 3 and 4), suggesting the association of Thr668 P-APP with degenerating neurites. Ntg mice and PS1 mice did not show any specific staining with either Thr668 P-APP or SMI34 antibodies (Figure 4, rows 1 and 2).

### APP phosphorylation and processing in AD transgenic mice

**Figure 5:**
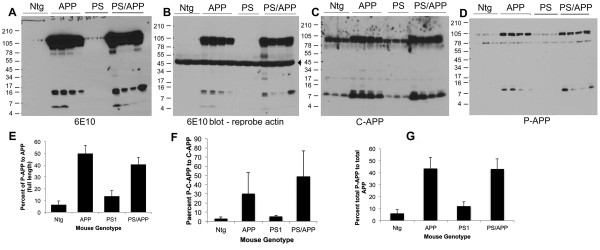
**Increased levels of APP phosphorylation and processing in transgenic mice expressing APP and PS/APP**: Equal amounts of proteins from Ntg, APP, PS1, and PS/APP brain extracts were analyzed using 6E10, C-APP, 22C11, and P-Thr668 APP antibodies. **A) **Shows western blot analysis using monoclonal 6E10 antibody (detect APP, Aβ, and any Aβ containing fragments of APP), **B) **shows reprobe of the same blot using actin antibody (indicated by arrow) without stripping to show equal amounts of protein loading, **C) **western blot using a polyclonal C-terminal APP antibody (detects full length and C-terminal fragments of APP), and **D) **represents the western blot using Thr668 P-APP antibody. Mice expressing APP and PS/APP showed very high levels of full length and C-terminal APP fragments. Aβ levels showed mouse-to-mouse variation probably due to varied expression of the transgenes. Levels of P-APP were significantly higher in both APP and PS/APP transgenic mice and the antibody detected the phosphorylated C-terminal fragment of APP as well. Blots were analyzed using supersignal ECL solution from Pierce. The histograms represent quantitative analysis of P-APP compared to the corresponding counterpart of total APP detected using C-terminal APP antibody: **E) **percent of full length P-APP, **F) **percent of P-C-APP (phosphorylated C-terminal fragment), and **G) **percent of total P-APP compared to total APP.

Western blot analysis of brain extracts from transgenic mice using the human specific 6E10 (raised against amino acids 1-16 of Aβ) antibody confirmed over expression of APP in both APP and PS/APP transgenic mice (Figure 5A). As 6E10 is more specific for human APP, for detection of mouse APP the blots were reprobed with C-APP antibody, which showed the total expression level of APP *in vivo *in the non-transgenic and transgenic mice (Figure 5C). The transgenic mice expressing APP and PS/APP showed very high levels of C-terminal APP fragments (Figure 5A and 5C). 6E10 antibody detected Aβ in both APP and PS/APP mice (Figure 5A). The level of Aβ showed variation between mice probably due to the altered levels of transgene expression. Examination of blots using Thr668 P-APP antibody showed that both full length and C-terminal fragment of APP show significantly higher levels of Thr668 phosphorylation compared to that detected in non-transgenic and PS1 mice (Figure 5D). This finding, along with the finding that in primary rat neurons the C-terminal fragment generated by BACE cleavage shows more phosphorylation at Thr668 than does α-secretase cleaved C-APP [42], suggests that the amyloidogenic cleavage of APP is enhanced upon phosphorylation at Thr668. The Thr668 P-APP antibody detects APP only when phosphorylated at Thr668 [40] and has been shown to react with human, mouse, and rat P-APP (Cell Signaling Technologies). On the western blot, it detected the intracellular levels of P-APP in the mouse (Ntg and PS), but the levels in APP expressing mice were significantly higher. The histograms in Figure 5E, F and 5G show the percent of P-APP (full length, C-terminal fragment, and total) compared to the total counterpart of APP detected by the C-terminal antibody, in the brain extracts.

### Age-dependent changes in Thr668 specific phosphorylation of APP in transgenic mice

**Figure 6:**
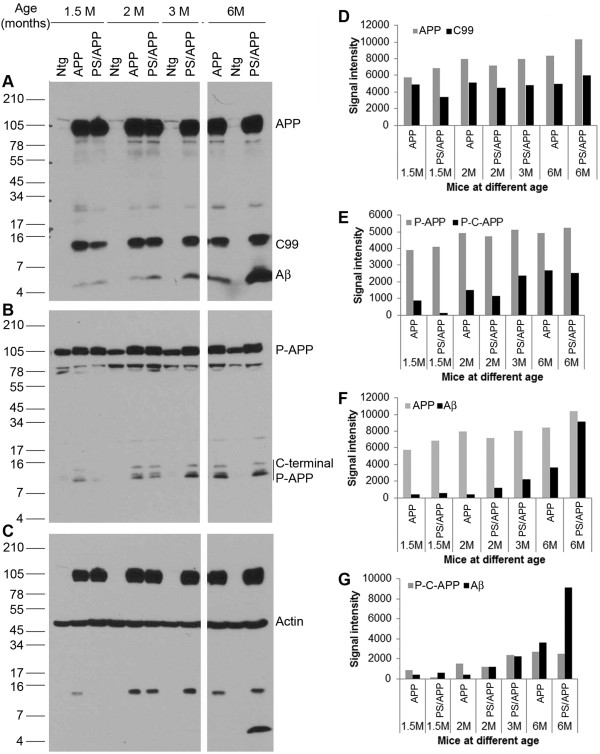
**Age-dependent changes in Thr668 specific phosphorylation and Aβ generation in transgenic mice**: Brain extracts from 1.5, 2, 3, and 6 month old Ntg mice and transgenic mice expressing APP and PS/APP were examined by western blot using Thr668 P-APP and 6E10 antibodies. Panel **A **shows the levels of full length APP and fragments of APP such as C-99 and Aβ in the mice at different ages. The transgenic mice expressing APP and PS/APP showed very high levels of full length APP. Only the levels of Aβ were altered in an age-dependent manner. Panel **B **shows staining of the blot with Thr668 P-APP antibody, which detects mouse and human APP phosphorylated at this site. The levels of full length P-APP were higher in the transgenic mice. Levels of phosphorylated C-terminal P-APP fragments were induced in an age-dependent manner in the transgenic mice. Panel **C **shows reprobe of the blot with actin antibody without stripping to show approximately equal amount of protein loading. **D-F **shows the relative signal intensity of the various APP fragments from the western blot analysis. **D**) Represents signal intensity of full-length APP and C-99 fragments, **E) **that of full length P-APP and phosphorylated C-terminal fragments of P-APP (P-C-APP), **F**) represents the levels of APP and Aβ and **G**) shows signal intensity of P-C-APP and Aβ.

The above results showed that APP phosphorylation and processing are enhanced in transgenic mice expressing APP. In order to determine whether the APP phosphorylation varies with age, we examined transgenic mice expressing APP and PS/APP at different ages. Brain extracts were prepared from mice at 1.5, 2, 3 and 6 months of age and western blotted using Thr668 P-APP and 6E10 Antibodies (Figure 6A-G). As expected, it was found that the total levels of APP and C99 fragments (using 6E10 antibody) were significantly higher in the AD transgenic mice (APP and PS/APP) compared to the non-transgenic mice. Within the transgenic groups, we did not observe any age-dependent increase in the levels of full length APP or C-99 fragments (Figure 6A and 6D). On the other hand, the transgenic mice showed an age-dependent increase in Aβ generation, with the mice expressing PS/APP showing higher levels of Aβ than that expressing just APP (Figure 6A and 6G). Expression levels of full length P-APP did not vary significantly with age whereas the levels of phosphorylated C-terminal fragments (P-C-APP) were increased in an age-dependent manner in the AD mice (Figure 6B and 6E). Figure 6F shows the levels of Aβ compared to full length APP in the transgenic mice, and Figure 6G shows the levels of Aβ compared to the levels of P-C-APP. A reprobe of the APP blots using actin antibody showed equal amounts of proteins on the blot (Figure 6C). It is known that pathology development in AD is age-dependent. The data presented here further demonstrates that APP phosphorylation and processing as well as Aβ generation are also age-dependent and these fragments may contribute to the enhanced pathology development in AD.

**Figure 7:**
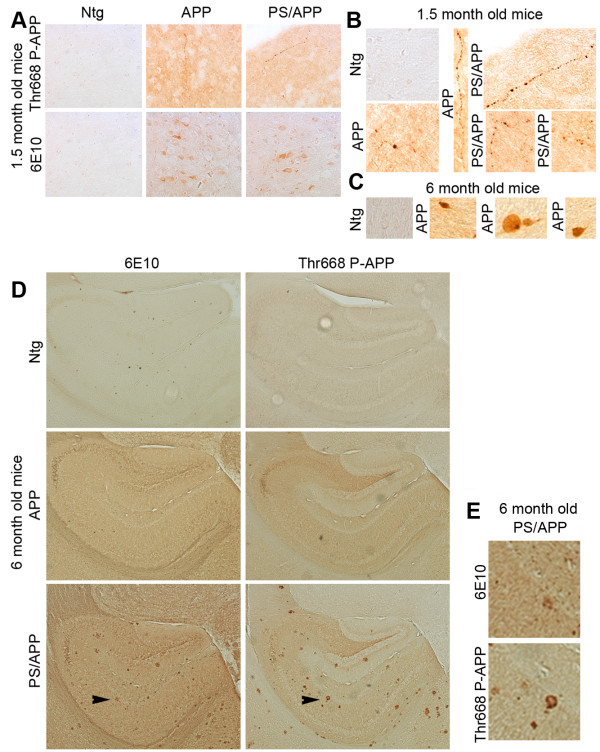
**Immunohistochemical analysis of brain sections from transgenic mice at different ages**: In order to determine whether there is accumulation of P-APP in the brain mice at 1.5 and 6 months were analysed using 6E10 and P-APP antibodies. Brain sections from transgenic mice showed an increase in overall staining using the 6E10 and P-APP antibodies **(A-D**). Panel **A **shows brain sections from 1.5 month old mice where P-APP showed beaded staining of neurites occasionally in APP and PS/APP mice. 6E10 staining showed APP in the neuronal bodies in these sections. The enlarged P-APP positive neurites are shown in panel **B. **Panel **C **shows examples of neurons in APP mice at 6 months that show P-APP accumulation. Panel **D **shows the P-APP and 6E10 staining in 6 month old Ntg, APP, and PS/APP mice. The accumulation of Aβ and P-APP are visible only in the PS/APP mice at 6 months. Panel **E **shows the magnification of the area shown with the arrows from 6E10 and P-APP stained PS/APP sections. Images in panel **A **were taken at 10× and in panel **B **at 20 × magnifications. Images shown in Panel **D **were taken at 5× magnification.

In order to determine the localization of P-APP in mice at different ages we examined the brains from 1.5 and 6 month old mice using Thr668 P-APP and 6E10 antibodies. 1.5 month old APP and PS/APP mice showed a general increase in overall staining and an association of P-APP with degenerating (beaded) neurites (Figure 7A, B shows enlarged images of beaded neurites in APP and PS/AP mice). 6E10 staining was mainly visible within the neuronal cell bodies of APP and PS/APP mice (Figure 7A). At 6 months of age the APP mice showed accumulation of P-APP in some neurons without any plaque pathology (Figure 7D, B shows neurons that show accumulation of P-APP). Unlike APP mice, 6 month old PS/APP mice showed very strong localized accumulation of P-APP in plaque-like structures, which did not always relate with 6E10 stained plaques (Figure 7D, E shows the enlarged image of the area indicated by arrows in PS/APP mice).

### Cell cycle-dependent phosphorylation of APP

**Figure 8:**
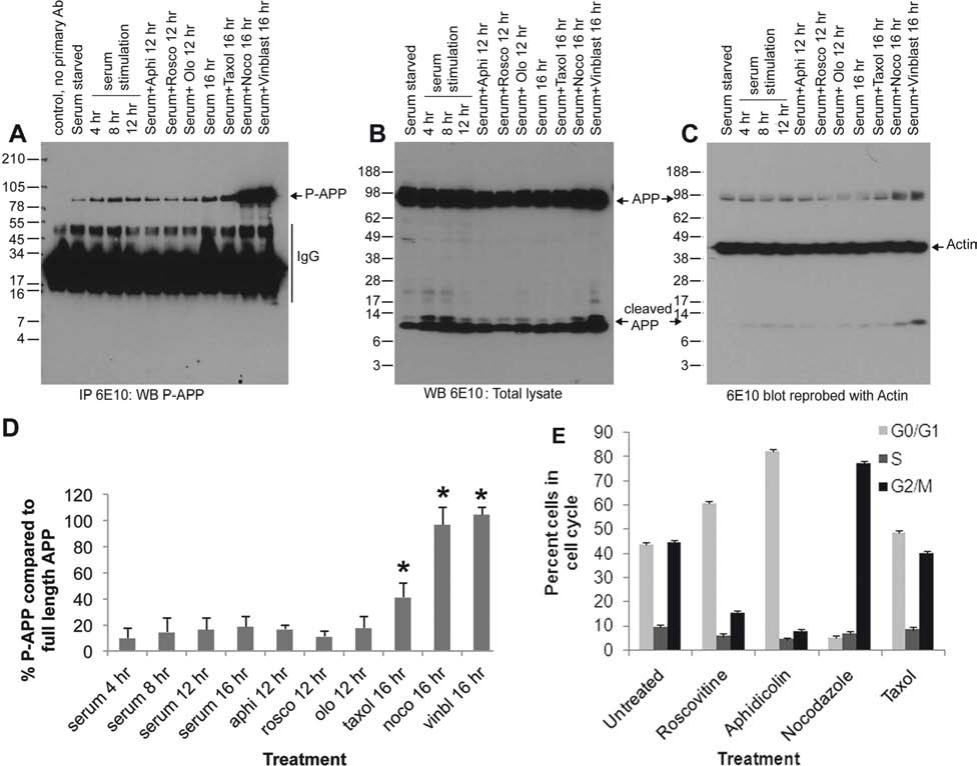
**Mitosis-specific phosphorylation of APP**: H4-15X cells were growth arrested by serum starvation for 48 hr and serum stimulated with and without roscovitine, olomoucine, or aphidicolin for 12 hr, and nocodazole, vinblastine, or taxol for 16 hr. Cell extracts were prepared and equal amounts of proteins were immunoprecipitated using 6E10 antibody and western blotted using P-APP antibody (**A**). **B**) APP levels in total lysate analyzed using 6E10 antibody. Panel **C **shows reprobe of blot B (without stripping) with actin antibody showing equal amount of proteins on gel. The histogram in panel **D **shows the percent of P-APP in cells under the different treatment conditions. The data represent the mean of 3 independent experiments with standard deviation shown. Cells arrested in metaphase showed significantly higher levels of P-APP (P < 0.05). Panel **E **shows the FACS analysis data from cells treated with cell cycle inhibitors. Cells were treated with roscovitine or aphidicolin for 12 hr or nocodazole or taxol for 16 hr and fixed and stained using propidium iodide before analysis on a FACS machine. Mean percent of cells in different phases of the cell cycle from 3 independent experiments is shown.

The phosphorylation of APP at Thr668 in transgenic mice correlated with the expression of cell cycle regulatory proteins in the brains. This result, together with the published findings [45], prompted us to determine whether the APP phosphorylation is due to cell cycle activation. Since it is difficult to verify this *in vivo*, we decided to examine APP phosphorylation in cells cultured *in vitro*. H4 neuroglioma cells overexpressing WT-APP (H4-APP) were cultured for 24 hr and serum starved for 48 hr. At the end of the starvation period, cells were serum stimulated in the presence or absence of pharmacological inhibitors of cell cycle progression for different time periods, and cell extracts were immunoprecipitated using 6E10 antibody and analyzed using P-APP antibody. The treatment of the cells included roscovitine (20 μM for 12-14 hr) an inhibitor of cdk2, cdc2, and cdk5 [52], olomoucine (50 μM for 12-14 hr) an inhibitor of cdk1 (cdc2), cdk2, and cdk5 [53], aphidicolin an S-phase inhibitor (5 μg/ml for 12-14 hr), or the mitotic inhibitors nocodazole (100 ng/ml), vinblastine (10 μM), or taxol (100 ng/ml) for 16 to 18 hr. Analysis of the cells using a fluorescence activated cell sorter (FACS) showed that roscovitine and aphidicolin treated cells were mostly arrested in the G1 phase of the cell cycle (60-70%), while nocodazole arrested cells were mostly in the G2/M phase (Figure 8E). Cell extracts after treatment were immunoprecipitated using 6E10 antibody and the blot was analysed using P-APP antibody. We found that Thr668 specific phosphorylation on APP was induced in a time dependent manner upon serum stimulation, and maximum levels of phosphorylation occurred upon mitotic arrest using nocodazole or vinblastine (Figure 8A and 8D). The total levels of APP (reprobe using 6E10 antibody) also showed a similar profile, but the levels were not as significant as those we observed with P-APP (Figure 8B). Reprobe of blots using actin antibody (without stripping) showed approximately equal amounts of proteins on the gel (Figure 8C). Roscovitine treatment consistently resulted in a decrease in the levels of P-APP, and aphidicolin and olomoucine kept the phosphorylation more or less at the basal levels (Figure 8A and 8D). These data confirm that the Thr668 specific phosphorylation on APP occurs in a cell cycle-dependent manner and peaks during mitosis. Cells treated for 16-18 hr with taxol, a microtubule stabilizing agent, largely accumulated in the G1 (~45-50%) and G2 (~40-45%) phases, which was similar to that observed with serum stimulation for 16 hr and showed slightly higher levels of P-APP compared to serum stimulated cells. The results from the time course with serum as well as treatment with roscovitine, olomoucine, and aphidicolin suggest that APP phosphorylation occurs very early during the cell cycle. Thus, it is possible that in the brains of transgenic mice APP phosphorylation arises from cells attempting to enter the cell cycle and inhibitors of G1/S checkpoint may inhibit this phenomenon.

### si-cdk2 inhibits serum stimulation-induced APP phosphorylation in H4-APP cells

**Figure 9:**
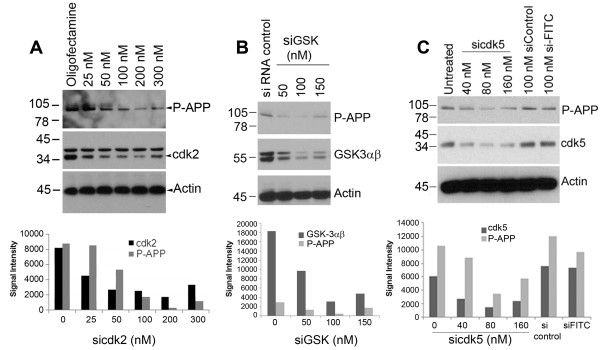
**siRNA to cdk2, cdk5, and GSK-3αβ inhibits serum stimulation-induced APP phosphorylation at Thr668**: H4-APP cells plated in serum-free OPTI-MEM were transfected with siRNA to cdk2 (Panel **A**), GSK-3αβ (panel **B**) or cdk5 (Panel **C**) at the indicated concentrations using oligofectamine. After 6 hr serum containing media was added to the cells and samples were collected after 24-48 hr. Cell lysates were western blotted using the corresponding kinase antibodies to confirm downregulation of the respective kinases. Phosphorylation status of APP was analyzed using P-Thr668 APP antibody, and actin was used as a loading control. Down regulation of each kinase was associated with inhibition of serum stimulation-induced phosphorylation on APP. The histograms below each blot show the quantification of the level of the respective kinase and P-APP compared to the levels present in siRNA control transfected cells. The data are representative of one of three independent experiments.

We found that APP is phosphorylated at Thr668 in a cell cycle-dependent manner and that roscovitine, an inhibitor of cdks such as cdc2, cdk2, and cdk5, prevented both APP phosphorylation and Aβ generation while nocodazole, a mitotic inhibitor, induced these phenomena. Earlier studies had shown that both cdk5 and cdc2 could induce Thr668 specific phosphorylation of APP while nothing was known about cdk2. In order to determine whether cdk2 or cdk4 are involved in APP phosphorylation during mitosis, we transfected H4-APP cells with different concentrations of cdk2 and cdk4 siRNA and analyzed for changes in APP after 16 to 18 hr. Western blot analysis of extracts using specific cdk antibodies confirmed the downregulation of the kinases in the transfected cells (Figure 9A). Analysis of extracts from cdk2 downregulated cells showed that this is associated with a decrease in APP phosphorylation at Thr668 (Figure 9A) whereas cdk4 inhibition was not (data not shown). A non-specific control siRNA did not have any effect on the kinases or APP phosphorylation, suggesting that the results we observed with si-cdk2 are specific to this cdk. In the G1 phase of the cell cycle, cdk2 associates with cyclin E and enables the transition of cells through the G1/S checkpoint. The result with si-cdk2 thus agrees with the results shown in Figure 8 in which APP phosphorylation is induced upon serum stimulation and is inhibited by blocking the G1/S transition.

Since it is known that cdk5 as well as GSK-3β can induce tau hyperphosphorylation in AD brains, and since these kinases have also been shown to affect APP phosphorylation, we examined the effect of downregulation of these kinases on APP phosphorylation. Cells transfected with siRNA to GSK-3αβ (Figure 9B) and siRNA to cdk5 (Figure 9C) also showed downregulation of APP phosphorylation as expected and it correlated with the levels of down regulation of the corresponding kinases. These data thus suggest that APP and tau are phosphorylated under similar conditions, and that inhibitors of these kinases should be tested for their ability to reduce development of pathology in AD. The G1/S inhibitor roscovitine has been shown to inhibit cdk5 and therefore the effect we see with this inhibitor could be due to its effect on not only cdk2 but other responsive kinases as well.

### Distribution of P-APP in asynchronously growing cells

**Figure 10:**
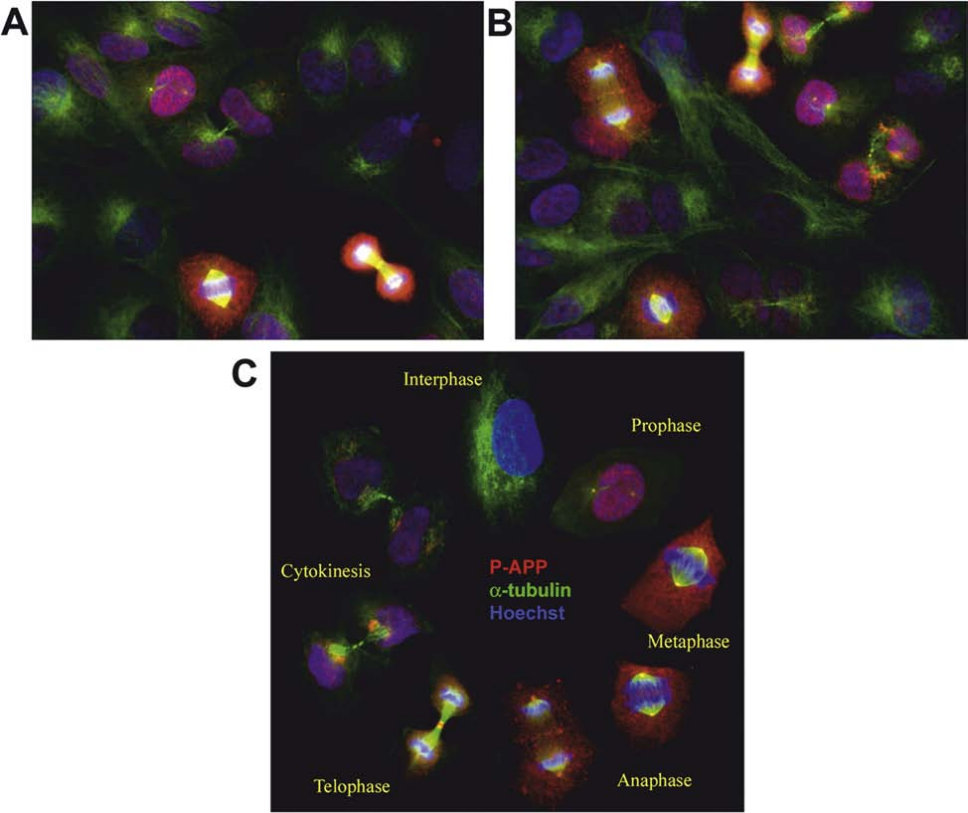
**Analysis of P-APP distribution in asynchronously growing H4-15X cells show cell cycle-dependent localization of P-APP**: Panels **A** and **B** show asynchronously growing H4-15X cells fixed and immunostained using Thr668 P-APP polyclonal and α-tubulin monoclonal antibodies and visualized using Alexa 594 (red) and 488 (green) fluorophores respectively. Staining was analyzed using the AxioVision Rel 4.8 software for Zeiss microscope. Nuclei were visualized using Hoechst staining. Cells in mitotic phase showed P-APP localized to the centrosomes, nucleus and cytoplasm with maximum immunoreactivity in mitotic cells and minimum/none in interphase cells. Cells in telophase showed P-APP staining in the midbody which was absent in cells undergoing cytokinesis. The absence of staining in the interphase cells suggests that APP phosphorylation at Thr668 occurs only when cells are undergoing division. Panel **C** shows a cell cycle schematic with representative cells from different stages of the cell cycle (selected from an asynchronously growing culture) illustrating the phosphorylation event occurring once the cells enter prophase and tapering off as it exits the cell cycle (cytokinesis). Magnification: 63×.

**Figure 11:**
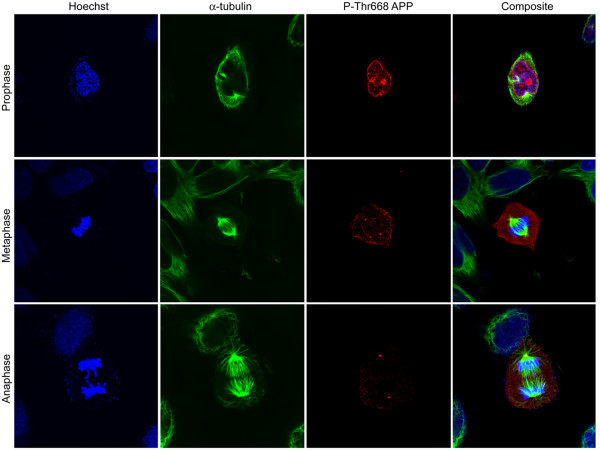
**Centrosome association of Thr668 P-APP in mitotic cells**: Asynchronously growing H4-APP cells were fixed and immunostained using Thr668 P-APP polyclonal and α-tubulin monoclonal antibodies and visualized using Alexa 594 (red) and 488 (green) fluorophores respectively. Nuclei were visualized using Hoechst staining. Staining was analyzed using the FV10-ASW 1.7 software for Olympus confocal microscope. Cells in metaphase and anaphase showed very clear P-APP localization at the centrosomes. Staining was very weak or absent in the interphase cells. Magnification: 63×.

Our results from H4-APP cells using the pharmacological inhibitors suggested that G1/S checkpoint inhibition prevents APP phosphorylation at Thr668. This prompted us to determine the expression and distribution of P-APP in cells at different phases of the cell cycle. First, we examined the localization of P-APP in asynchronously growing H4-APP cells. The cells were trypsinized and cultured for 24 hr and fixed and analyzed with monoclonal α-tubulin and polyclonal Thr668 P-APP antibodies. As expected, the asynchronously growing culture contained a non-homogeneous population of cells in different phases of cell cycle (Figure 10A and 10B). P-APP showed a cell-cycle specific localization with more staining in cells that are in active division (prophase, metaphase, anaphase) and very little or no staining in early interphase cells (Figure 10A-10C). The cells that were in metaphase showed prominent localization of P-APP to the centrosomes (microtubule organizing centers; MTOCs) suggesting a role for phosphorylated APP in cell cycle activation and/or spindle assembly. We confirmed the localization of P-APP to centrosomes in metaphase cells using confocal microscopy (Figure 11). Cellular distribution of P-APP was also examined in cells treated with the pharmacological inhibitors roscovitine, aphidicolin, nocodazole, or taxol. While cells treated with roscovitine and aphidicolin showed very few cells in active division and hence little P-APP staining, the majority of the nocodazole treated cells were in the mitotic phase and exhibited significantly higher levels of P-APP (data not shown).

### Evidence for mitotic phosphorylation and centrosome localization of P-APP

**Figure 12:**
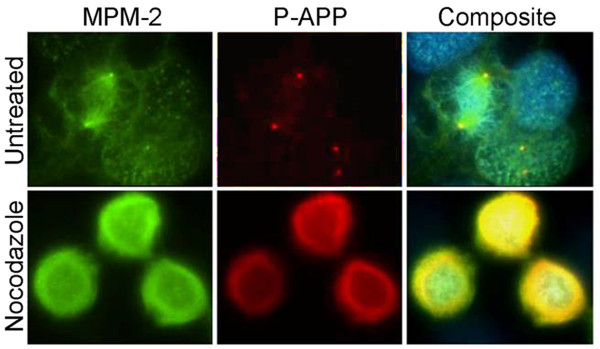
**P-APP co-localization with MPM-2 at centrosomes in metaphase cells**: Asynchronously growing (untreated, top row) and nocodazole arrested (bottom row) H4-15X cells were immunostained using the mitosis specific monoclonal antibody MPM2 and Thr668 P-APP polyclonal antibodies and staining was visualized using Alexa 488 and 594 fluorophores respectively. The untreated cells show P-APP localization in centrosomes in the mitotic cells. In the cells arrested with nocodazole the microtubules were completely depolymerized and P-APP showed significantly higher levels of amorphous staining. The nuclei were visualized using Hoechst stain. Magnification: 63×.

In order to confirm that the phosphorylation of APP at Thr668 is mitosis-specific, we co-stained cells with antibodies that are specific for mitotic phosphoepitopes. The best-characterized antibodies for this purpose are the metaphase protein monoclonal-2 (MPM2). MPM2 antibodies detect phosphoproteins that are present in mitotic cells and have been shown to associate with the kinetochore, centrosome, midbody and fibers of the mitotic spindle [54]. Our analysis of asynchronously growing untreated cells showed that, in metaphase, P-APP co-localized with MPM2 at the centrosomes (Figure 12 top row). In cells arrested with nocodazole, although P-APP levels were significantly elevated (Figure 12 bottom row), due to the microtubule destabilizing function of nocodazole we did not observe any metaphase cells with classic spindles and spindle poles. The high levels of P-APP in these cells correlated well with the western blot data and confirm that APP is heavily phosphorylated under mitotic conditions. These results suggest that the phosphorylation of APP may play a role in cell cycle dependent processes including centrosome replication.

### Cell cycle activation induces altered processing of APP and Aβ generation

**Figure 13:**
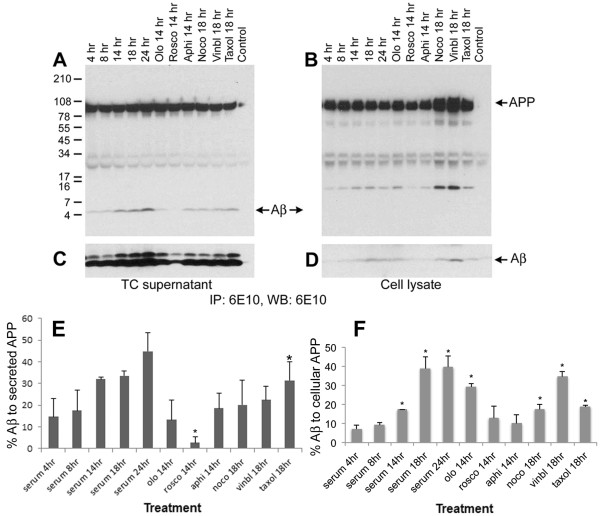
**Aβ generation is altered in a cell cycle-dependent manner**: H4-15X cells were synchronized by serum starvation and stimulated with serum containing media plus or minus olomoucine, roscovitine, aphidicolin, nocodazole, vinblastine, or taxol. Cell culture supernatants (**A** and **C**) and cell extracts (**B** and **D**) were immunoprecipitated and western blotted using 6E10 antibody. Control was performed similarly to the rest of the samples, except no primary antibody was used in immunoprecipitation assay. Cell culture supernatant showed a time dependent increase in Aβ generation upon serum stimulation (**A** and **C**). The extracts showed similar results which was visible only after longer exposure (**D**). Panels **C** and **D** represent longer exposure of the bottom part of the blots shown in **A **and **B **to show the Aβ levels in cell extracts. In both the cases roscovitine treatment was associated with a decrease in the level of secreted and cellular Aβ. Secreted APP was not altered in the supernatant although the level of APP and C-APP fragments are increased in the extracts prepared from nocodazole and vinblastine-arrested cells. Panel **E **and **F** show mean percent of Aβ (compared to secreted and full length APP) from 3 independent experiments under different treatment conditions. Data that showed significant changes are marked using a star (P < 0.05).

We next examined whether Aβ generation is altered in a cell cycle-dependent manner and whether it parallels the phosphorylation of APP. Cells were growth arrested by serum starvation for 48 hr and serum stimulated in the presence or absence of pharmacological inhibitors of cell cycle progression for different time periods. The cell culture supernatants and cell extracts were then immunoprecipitated and western blotted using the Aβ-specific 6E10 antibody. The results showed that intracellular and secreted levels of Aβ increase in a time-dependent manner upon serum stimulation of growth arrested cells (Figure 13A-D, C and 13D show the bottom region of the blot from longer exposure to detect Aβ in lysates), and followed a similar profile as in APP phosphorylation (Figure 8). The histograms in E and F show the percent levels of Aβ in supernatant and lysate in comparison to the total levels of full length APP in the respective samples. In the case of cells arrested with pharmacological agents, the maximum levels of intracellular and secreted Aβ were present in mitosis-arrested cells (nocodazole, vinblastine, and taxol) with the lowest levels in the roscovitine treated (G1/S inhibited) cells. Treatment of neurons in vitro with Aβ peptide has been shown to induce cell cycle activation and neuronal apoptosis [27,49]. Thus, these findings not only imply that APP is phosphorylated and processed in a cell cycle-dependent manner, but also suggest that the observed cell cycle activation in the brains of AD transgenic mice may induce APP phosphorylation, leading to enhanced levels of intracellular and extracellular Aβ that subsequently induce cell cycle activation and neurodegeneration.

Upon analysis of other proteolytic fragments of APP in cultured cells, we found that, similar to our observations in AD transgenic mice, phosphorylation was associated with increased BACE cleavage of APP, as evident by the C-terminal fragment detected by the 6E10 antibody. The C-APP levels were lowest in roscovitine treated G1/S checkpoint arrested cells. Recent studies using a C-terminal fragment of APP have shown that the administration of this fragment induces apoptosis in cells of neuronal origin [55]. Thus, our results support the suggestion that inhibitors of G1/S transition may prevent neurodegeneration by preventing unwarranted processing of APP to generate neurotoxic Aβ and C-APP.

## Discussion

Alzheimer's disease is characterized by the presence of neuritic plaques and neurofibrillary tangles in the affected areas of the brain. In addition, AD brains show considerable neuronal loss and neuroinflammation, the causal mechanisms of which are under active investigation. Studies from several laboratories have shown that AD brains exhibit aberrant upregulation of cell cycle regulatory proteins [4,6,7,14,22,56]. It is suggested that the deregulated expression of cell cycle proteins in neurons may contribute to the pathology associated with Alzheimer's, possibly due to inappropriate induction of the cell cycle in post-mitotic neurons. A causal link can be established between cell cycle activation, neurodegeneration, and neuronal loss *in vitro*, but it has been difficult to illustrate how cell cycle activation can induce a slowly developing but ultimately catastrophic effect in human AD brain. In order to understand the mechanisms involved in cell cycle activation and AD pathogenesis, we used mice expressing APP_V717F _and PS1_M146L _mutant transgenes. The PS/APP mice develop plaques at approximately 6 months of age and the APP mice show plaques at approximately 10-12 months of age. We found that, similar to human AD, brains from these mice also show increased expression of some of the cell cycle regulatory proteins. This was associated with increased phosphorylation of APP.

*In vitro *analysis of asynchronously growing H4-APP cells clearly showed that the phosphorylation of APP occurs mainly in the cells that are undergoing cell division. In the interphase, cells APP phosphorylation was negligible and was induced as soon as the cells entered prophase. The experiments with si-cdk2 and pharmacological inhibitors of the G1/S checkpoint further supports the conclusion that APP phosphorylation and processing occurs in a mitosis-specific manner and reinforces the idea that inhibition of cell cycle activation at an early stage may prevent the APP modifications associated with the development of AD pathology. APP phosphorylation is not just mediated by cyclin-dependent kinases. Kinases such as GSK-3β, JNK, and cdk5 have also been shown to affect Thr668 specific phosphorylation of APP. Our studies also showed that this specific phosphorylation could be inhibited by downregulation of GSK-3β and cdk5. Both GSK-3 and cdk5 have been shown to play roles in the cell cycle and hence the possibility that these kinases are also behaving in a cell cycle-dependent manner needs to be established [57,58]. Nocodazole-induced mitotic arrest led to a significant increase in APP phosphorylation compared to that induced by serum stimulation alone. One of the reasons for this result could be that the number of metaphase cells obtained upon treatment with nocodazole (~80% by FACS analysis) is much higher than that obtained by serum stimulation or taxol treatment (~40%). The data shown in Figure 8 agrees with this interpretation; quantitative analysis of the levels of P-APP and APP showed that while serum stimulation shows ~30% APP phosphorylation (~40% cells in G2/M), nocodazole treatment shows ~80%, both of which correlate with the percent of cells in metaphase. In addition, nocodazole, being a powerful microtubule depolymerizing agent, could affect other kinases or phosphatases and induce APP phosphorylation independent of its mitotic arrest-related effects. Treatment of cells with taxol, another mitotic inhibitor that brings about cell cycle arrest through microtubule stabilization, showed only ~40% of cells in metaphase and a P-APP level of ~30%.

The results presented here strongly indicate that Thr668 specific phosphorylation on APP is intimately associated with cell cycle activation and that the maximum phosphorylation occurs in metaphase. This phosphorylation transition was associated with increased APP processing and Aβ generation (Figure 13). Thus the cells do not have to go through a full division to bring about the modifications in APP suggesting that an attempt by the cells in AD brain to re-enter cell cycle could lead to APP phosphorylation and proteolytic cleavage without the cells undergoing cell division. The findings that AD brains show binucleated neurons [59], as well as aneuploidy and mis-segregation of different chromosomes [15,61-63] further strengthens the conclusion that neurons in AD brain attempt to undergo DNA replication and cell division. It is suggested that the cell cycle regulatory proteins may have a different role in neurons compared to that in cells undergoing active cell division; studies show that terminally differentiated neurons use the mechanisms involved in proliferation to maintain the synaptic plasticity [42,60,64]. It is possible that the complex architecture of mature plastic neurons makes it impossible for the cells to undergo division without undergoing damage. The report that centrosomes localize to the area where the neurites sprout from and the number of centrosomes determines the number of neurites [65] suggest that cell cycle activation may cause asymmetric dynamics on the chromosomes leading to mis-segregation and formation of aneuploid cells in the AD brain. The localization of P-APP (data presented here) and PS1 at the centrosomes [66] suggest that these molecules may play a role in spindle assembly and chromosome segregation, and hence the enhanced expression or mutations of these proteins may cause chromosome mis-segregation in cells. The data from our lab support this hypothesis, in which we showed that expression of APP, Aβ, or PS1 lead to chromosome mis-segregation and aneuploidy [36]. Aβ oligomers have been reported to induce neuronal cell cycle activation [49,50], and this along with the data presented here, suggest that Aβ generated upon APP phosphorylation may have a feed forward role in cell cycle activation and enhanced neurodegeneration in AD brain.

Cell cycle activation not only induced the phosphorylation and proteolytic processing of APP, but also affected the localization of P-APP in cells; mitotic cells clearly showed centrosome specific localization of P-APP. It has been proposed that the phosphorylation of structural or transient components of centrosomes may affect cell cycle dependent processes such as centrosome duplication and microtubule nucleation [54]. Thus, in addition to enhanced proteolytic cleavage, APP phosphorylation may influence cell proliferation through its association with the cell cycle machinery. The co-localization of P-APP with MPM2, a metaphase protein marker, further reiterates APP's role as a growth-promoting molecule. Therefore, it is possible that high levels of P-APP may promote proliferation in dividing cells and centrosome duplication or chromosome mis-segregation and cell death in post-mitotic neurons. APP's function as a mitogenic molecule is evident from the fact that its upregulation is associated with cancers of different organs [67,68]; neurons being postmitotic are fully differentiated and undergo apoptosis rather than transformation upon cell cycle activation. It has been reported that APP and PS1 associate with other proteins at the centrosome and localize to centrosomes [69,70]. An N-terminal APP antibody conjugated to an Alexa fluorophore was used to detect the localization of APP at the centrosomes. In our hand staining of the cells with the Aβ immunoreactive 6E10 antibody did not show any significant localization of non-phospho APP to the centrosomes. It is possible that either the antibody or the techniques we applied to detect the localization are not strong enough to detect non-phospho APP at the centrosomes.

Our studies showed that APP and PS/APP mice show formation of Aβ and phosphorylated C-terminal fragments of APP at a very early age (1.5 month), and the generation of these fragments are increased in an age-dependent manner. Although this is the case, PS/APP mice showed clear accumulation of P-APP and Aβ in their brains by 6 months of age whereas APP mice showed only by 10-12 months. This result suggests that unless there is an accelerating factor present, the pathology in AD develops very slowly and if diagnosed early in life it can be prevented. The facts that deregulation in PS1 can induce chromosome miss-segregation and tumour generation [36,71], and both PS1 and APP associate with centrosomes, suggest that in addition to Aβ generation, expression of PS1 in APP transgenic mice may affect cell cycle deregulation and therefore APP phosphorylation. Whether or not these associates with early neurodegeneration and neuronal loss observed in AD brains needs to be determined. Even though the PS/APP mice we used show significantly higher levels of P-APP and accumulation of Aβ in to plaques, unlike in some of the other AD mouse models such as APP^SL^/PS1-KI and 5XFAD mice [68,72,73], we did not observe any significant neuronal loss. The reason for this is unclear. It is possible that the genetic background and the transgene expression levels play a role in plaque load and neuronal loss associated with different transgenic mouse models. The APP mice we used do not exhibit as aggressive an AD-like disease as the ones above, and probably inclusion of an additional APP mutation in the transgene may be required to obtain detectable levels of neuronal loss.

## Conclusions

In conclusion, cell cycle deregulation may influence the pathogenesis of AD through multiple pathways: 1) through phosphorylation and processing of APP to generate Aβ leading to plaque formation, 2) through Aβ and C-terminal fragment of APP inducing tau hyperphosphorylation [66,74-76], and 3) through both Aβ and P-APP affecting cell cycle deregulation and contributing to the unwarranted progression of cell cycle. From the data presented here it is apparent that an inhibition of aberrant activation of the cell cycle prior to G1/S checkpoint could potentially hinder the modifications in APP and therefore development of AD pathology. In this respect, G1/S inhibitors, which are known to protect neuronal apoptosis *in vitro *[26,31,32], need to be explored *in vivo *for their efficacy in preventing APP phosphorylation and processing. Once the neurons start expressing higher levels of cell cycle proteins due to environmental stress, or inflammation, or high levels of Aβ, the modifications in the proteins associated with the development of pathology will take place, and the cells will succumb to degeneration. The data presented above and the previous support for the cell cycle hypothesis, which suggests that the neurons in AD brain enter the G1 phase of cell cycle [6,7,77], indicate that inhibitors of the early phases of cell cycle such as those associated with the G1/S checkpoint may prove to be beneficial in treating neurodegenerative diseases such as Alzheimer's. However, it must be noted that microtubules are essential for many neuronal functions, and thus any drugs designed to inhibit APP modifications or Aβ generation should be tested for their effect on microtubule dynamics both *in vitro *and *in vivo *before assuming that they will be risk-free therapies for AD.

## Methods

### Ethics Statement

All studies involving animals were done in accord with the rules and regulations set forth by the University of South Florida's Institutional Animal Care and Use Committee (IACUC). The care for the animals was provided by the well-established animal care facility at University of South Florida (USF), which is accredited by the American Association of Laboratory Animal Care (AALAC).

### Materials

The tissue culture reagents, electrophoresis supplies, and Alexa fluorphores were purchased from Gibco/Invitrogen, Carlsband, CA. Poly-D-Lysine (PDL), α-tubulin antibody and Hoechst were from Sigma, St. Louis, MO. Anti-Aβ/APP antibody (6E10 raised against Aβ1-16) was from Signet, C-terminal APP antibody was from Chemicon/Millipore, Thr668 P-APP, MPM-2, and P-cdc2 antibodies were from Cell Signaling, and cyclin D1, cyclin E, and E2F1 antibodies were from Santa Cruz Biotechnology. The reagents for brightfield staining were purchased from Vector Laboratories. Enhanced chemiluminescence (ECL) reagent was from Pierce Biotechnology Inc., Rockford, IL. H4 neuroglioma cells overexpressing WT-APP (H4-APP) was a kind gift from Dr. Todd Golde (Mayo clinic, Jacksonville, Florida).

### Transgenic Mice

Heterozygous PDGF-hAPP (V717F) mice (Swiss-Webster × C57BL/6) were crossed with PDGF-hPS1 (M146L) heterozygotes (Swiss-Webster × C57BL/6) to generate mice with an APP^+/-^, PS1^+/- ^genotype. All offspring were screened by PCR to verify the expression of APP and PS1 gene [78,79]. The APP mutant mice develop many of the pathological hallmarks of AD, including neuritic plaques (appear at around 10-12 months of age), and cognitive deficits in an age-dependent manner, and the expression of mutant PS1 in these mice accelerates the pathology development significantly (plaques are visible as early as 4-6 months of age) (Figure 7D). In the current study we used these transgenic and age-matched non-transgenic (Ntg) mice. Mice were anesthetized using Nembutal (10 mg/kg body weight) and perfused with saline solution. The brains were dissected out and half of each brain was immersion fixed with 4% para-formaldehyde and the other half was used for protein extraction. For protein extraction, brains were homogenized in Hepes lysis buffer (50 mM HEPES pH 7.4, 150 mM NaCl, 10% glycerol, 1% Triton X-100, 5 mM MgCl_2_, 1 mM EGTA, 20 mM NaF, 2 mM Na_3_VO_4_, and protease inhibitors (Roche)). Samples were centrifuged at 14,000 rpm for 30 min and equal amounts of proteins were used for western blot analysis. The brains were processed as described before for immunohistochemical analysis [80]. Brain sections were made using a freezing stage sliding microtome and stored at 4°C in phosphate buffered saline (PBS) containing sodium azide (0.02%) for immunohistochemical analysis.

### Immunostaining

This was done following the established protocols [80,81]. Briefly, H4-15X cells cultured in 8-chamber tissue culture slides coated with PDL were treated with or without different inhibitors of the cell cycle for 18 hr; roscovitine (20 μM) as G1/S inhibitor, aphidicolin (5 μg/ml) as S-phase inhibitor, nocodazole or vinblastine (100 ng/ml or 10 μM respectively) or taxol (placitaxel, 100 ng/ml) as mitotic inhibitor. At the end of the treatment, cells were fixed with 4% para-formaldehyde and staining was performed using the appropriate antibodies. Staining was analyzed under a Zeiss microscope using the AxioVision Rel 4.8 software. Centrosome specific staining of P-APP in H4-APP cells was confirmed by confocal microscopy under an Olympus imaging system using Fluoview FV1000 ver.1.7 software.

For immunostaining analysis of the brain sections, sections were mounted onto superfrost slides, and non-specific binding was blocked by incubating with 10% normal goat serum (NGS)/TBST for 2 hr at room temperature. Sections were then incubated with appropriate dilutions of the primary antibody (APP (6E10), Thr668-P-APP, cyclin D1, cyclin E, E2F1, and P-cdc2 antibodies) in 1% BSA/TBST overnight at 4°C in a humidified chamber. After thorough washing, the sections were incubated with biotinylated mouse or rabbit secondary antibodies for 1 hr at room temperature and developed following the manufacturer's protocol with the DAB kit from Vector laboratories. The staining was visualized using a Nikon E1000 microscope using Image-Pro Plus software. In the case of fluorescent labeling, after primary antibody incubation as outlined above, sections were incubated with Alexa 488 or 594 fluorophores for 2 hr at room temperature protected from light. Sections were washed and nuclei were counter stained using Hoechst 33342 and washed again before mounting using aqueous Gel/Mount. Sections were stained with secondary antibody alone to determine non-specific binding of antibodies to the tissue (data not shown). The results were analysed under a Zeiss microscope using the AxioVision Rel 4.8 software. The signal intensity of the images was determined by Image J, image processing and analysis program [32]. Adjacent sections from at least 3 independent mice expressing different transgenes were stained using the antibody of interest. Prior to measurement, the images were converted to 8-bit grayscale and the threshold of all the images from each set of experiments was adjusted to the same level. This keeps the sample-to-sample variation minimal. The intensity obtained with Hoechst staining was used as a normalizing control for each section.

### Immunoprecipitation and Western blot analysis

H4-15X cells were cultured in OPTI-MEM containing 10% FBS and 50 μg/ml hygromycin in 100 mm culture dishes overnight and serum starved for 48 to 72 hr. Serum stimulation of the cells was done in the presence or absence of different pharmacological inhibitors for the indicated time periods. Cell culture supernatants and cell lysates (made in Hepes lysis buffer) were immunoprecipitated using 6E10 antibody and analyzed using the same antibody to detect secreted and cellular levels of Aβ. In the case of brain extracts, equal amounts of protein were boiled with Tricine sample buffer and PAGE and western-immunoblot analysis was performed using appropriate antibodies. For quantification, western blot images on the X-ray film were scanned and densitometric analysis was performed using the Image J, image processing and analysis tool after selecting and plotting the bands of interest.

### siRNA transfection of H4-APP cells

We obtained Silencer validated siRNA to cdk2 (locus ID: 1017) from (Ambion, Inc. Applied Biosystems), siRNA to cdk5 from Santa Cruz Biotechnology and siRNA to GSK-3αβ from Invitrogen. The siRNA was used at the indicated concentrations and transfected using oligofectamine (Invitrogen) using OPTI-MEM without serum. 6 hr after transfection the media was replenished with an equal volume of OPTI-MEM containing 2X serum and cultured for 24 to 48 hr. At the end of the time period cells were harvested in sample buffer and analyzed by western blot for downregulation of the kinases using the corresponding kinase antibody and phosphorylation of APP by Thr668 P-APP antibody.

Statistical analysis was performed using Student's t-Test.

## List of abbreviations

AD: Alzheimer's disease; APP: amyloid precursor protein; PS: presenilin; Thr668 P-APP: APP phosphorylated at Threonine 668; C-APP: C-terminal APP; P-C-APP: phosphorylated C-APP; cdk: cyclin-dependent kinase.

## Competing interests

The authors declare that they have no competing interests.

## Authors' contributions

JP designed and carried out all of the in vitro studies and part of the in vivo studies, ML was instrumental in the cell cycle analysis of transgenic brains, LH helped with western blot analysis of brain extracts, and HP provided the transgenic mice and helped with scientific discussions. All authors have read and approved the final manuscript.
